# The Influence of Conjugated Polymer Side Chain Manipulation on the Efficiency and Stability of Polymer Solar Cells

**DOI:** 10.3390/ma9030181

**Published:** 2016-03-09

**Authors:** Ilona M. Heckler, Jurgen Kesters, Maxime Defour, Morten V. Madsen, Huguette Penxten, Jan D’Haen, Bruno Van Mele, Wouter Maes, Eva Bundgaard

**Affiliations:** 1 Department of Energy Conversion and Storage, Technical University of Denmark (DTU), Frederiksborgvej 399, Roskilde 4000, Denmark; ilhe@dtu.dk (I.M.H.); mves@dtu.dk (M.V.M.); 2 Institute for Materials Research (IMO-IMOMEC), Hasselt University, Agoralaan 1-Building D, Diepenbeek 3590, Belgium; jurgen.kesters@uhasselt.be (J.K.); huguette.penxten@uhasselt.be (H.P.) jan.dhaen@uhasselt.be (J.D.H.); wouter.maes@uhasselt.be (W.M.); 3 Physical Chemistry and Polymer Science (FYSC), Vrije Universiteit Brussel (VUB), Pleinlaan 2, Brussels 1050, Belgium; mdefour@vub.ac.be (M.D.); bvmele@vub.ac.be (B.V.M.)

**Keywords:** conjugated polymers, side chain variation, organic photovoltaics, roll-coating, photochemical and thermal stability

## Abstract

The stability of polymer solar cells (PSCs) can be influenced by the introduction of particular moieties on the conjugated polymer side chains. In this study, two series of donor-acceptor copolymers, based on bis(thienyl)dialkoxybenzene donor and benzo[*c*][1,2,5]thiadiazole (BT) or thiazolo[5,4-*d*]thiazole (TzTz) acceptor units, were selected toward effective device scalability by roll-coating. The influence of the partial exchange (5% or 10%) of the solubilizing 2-hexyldecyloxy by alternative 2-phenylethoxy groups on efficiency and stability was investigated. With an increasing 2-phenylethoxy ratio, a decrease in solar cell efficiency was observed for the BT-based series, whereas the efficiencies for the devices based on the TzTz polymers remained approximately the same. The photochemical degradation rate for PSCs based on the TzTz polymers decreased with an increasing 2-phenylethoxy ratio. Lifetime studies under constant sun irradiance showed a diminishing initial degradation rate for the BT-based devices upon including the alternative side chains, whereas the (more stable) TzTz-based devices degraded at a faster rate from the start of the experiment upon partly exchanging the side chains. No clear trends in the degradation behavior, linked to the copolymer structural changes, could be established at this point, evidencing the complex interplay of events determining PSCs’ lifetime.

## 1. Introduction

**Figure 1:**
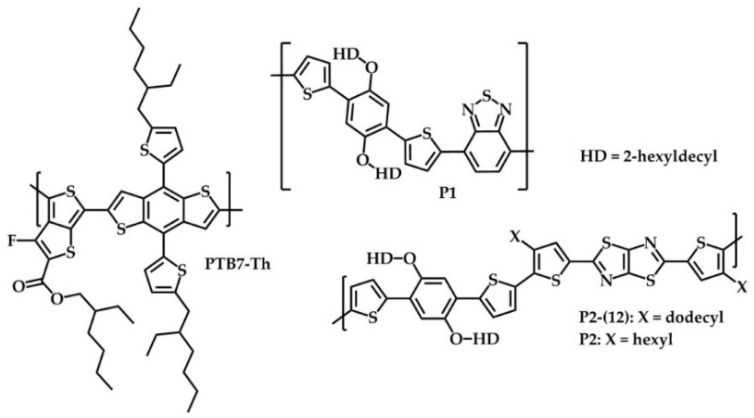
Chemical structures of a few low bandgap copolymer organic photovoltaics (OPV) reference materials [14,15,16]. PTB7-th, poly{4,8-*bis*[5-(2-ethylhexyl)thiophen-2-yl]benzo[1,2-*b*:4,5-*b*′]dithiophene-2,6-diyl-*alt*-3-fluoro-2-[(2-ethylhexyl)carbonyl]thieno[3,4-*b*]thiophene-4,6-diyl}.

Polymer solar cells (PSCs) have emerged as strong competitors in the field of renewable energy over the past two decades. Dedicated studies have highlighted their potential and wide applicability due to a myriad of inherent appealing features (low weight, flexibility, semi-transparency, color-tunability, *etc*.) [1,2,3,4,5]. Judicious efforts on chemical engineering of the polymer (electron donor) materials, optimization of the solar cell architecture and the acquisition of additional fundamental insights on device operation have driven the power conversion efficiencies (PCEs) of this technology to levels approaching and even surpassing the 10% threshold [6,7,8]. On the downside, some of the highly advanced conjugated polymers, such as PTB7-Th (poly{4,8-bis[5-(2-ethylhexyl)thiophen-2-yl]benzo[1,2-*b*:4,5-*b*′]dithiophene-2,6-diyl-*alt*-3-fluoro-2-[(2-ethylhexyl)carbonyl]thieno[3,4-*b*]thiophene-4,6-diyl}; Figure 1), require rigorous processing procedures (inert atmosphere, high temperature, *etc*.) and have so far only been successful on small laboratory-scale spin-coated (SC) devices [9]. However, for PSCs to become economically viable, large-scale production techniques (such as roll-coating (RC)) are mandatory [10,11]. RC processing has been applied for several years within the group at the Technical University of Denmark, but a recent screening of a myriad of polymers demonstrated only a few suitable candidates for large-scale (RC) device fabrication [12]. As an example, PTB7-Th, very promising in SC devices with PCEs up to 10% [13], did not allow for a proper translation to RC processing and only granted a PCE of 0.2% [12]. On the other hand, with respect to large-scale device fabrication, poly{[2,5-bis(2-hexyldecyloxy)phenylene]-*alt*-[4,7-di(thiophen-2-yl)benzo[*c*][1,2,5]thiadiazole]} **P1** and poly{2,2′-[5,5′-(2,5-bis(2-hexyldecyloxy)-1,4-phenylene)dithiophene]-*alt*-[2,5-bis(4-hexylthiophen-2-yl)thiazolo[5,4-*d*]thiazole]} **P2** (Figure 1) have been identified as suitable donor polymers for RC-processed organic photovoltaics (OPV) [12,14]. The moderate efficiency of the PSCs based on the **P2**-(**12**) polymer, containing the bis(thienyl)di(2-hexyldecyloxy)benzene donor part [12], could be improved using a hexyl side chain on the dithienylthiazolo[5,4-*d*]thiazole group (instead of dodecyl) [15]. In previous work on **P1**, the impact of the nature (alkyl/alkoxy) and positioning of the appended side chains on the electrical properties was demonstrated, especially with regards to the absorption profile, device performance and stability [16].

Since the introduction of the bulk heterojunction concept, in which the electron donating (polymer or small molecule) and electron accepting (often (methano)fullerene) components are intimately intermixed at the nanoscale within the photoactive layer, the morphology formation and evolution of the photoactive blend, processed from organic solvents, has been a fascinating and highly important topic in the field. It has been well described that the appended side chains on conjugated polymers have a large impact on both material solubility and blend formation [17,18]. On the other hand, over time, phase separation of the blend components can occur, especially upon the application of (external) stress (heat, light, *etc*.) [19]. The use of nucleation agents [20], non-crystallizing polymer-fullerene blends [20,21], crosslinkable functional moieties [22] and post-deposition removal of the side chains [23,24] has been proposed as potential routes to circumvent these issues and increase the lifetime of the solar cell devices fabricated from these materials. Recently, it was also shown that decreasing the side chain density of a 4*H*-cyclopenta[2,1-*b*:3,4-*b*′]dithiophene (CPDT)-based low bandgap copolymer afforded a synergetic enhancement of solar cell performance and thermal stability [25]. In addition, past efforts have indicated that full displacement of the dimethyloctyloxy side chains on MDMO-PPV (poly[2-methoxy-5-(3′,7′-dimethyloctyloxy)-1,4-phenylenevinylene]) to 2-phenylethoxy (EtPh) moieties resulted in a significant enhancement of the glass transition temperature (*T*_g_; from 45–111 °C) and, therefore, the thermal stability of the resulting PSCs, attributed to a reduced phase separation tendency of the active layer components [26]. A similar active layer stabilizing effect was obtained upon incorporation of small amounts (5%–10%) of alcohol or ester functionalities on the side chains of P3HT or PCPDTBT copolymers [27,28].

In this work, the aforementioned strategies were translated onto two promising RC processable conjugated polymers (**P1** and **P2**; Figure 1), on which the appended solubilizing 2-hexyldecyloxy (HD) side chains were partially exchanged by EtPh groups, in an attempt to elevate the *T*_g_ and, thereby, the (thermal) stability of the resulting photovoltaic devices. The influence of this side chain variation on the photovoltaic performance, as well as the thermal and photochemical stability (and thus, the lifetime) of the resulting solar cell devices was investigated.

## 2. Results and Discussion

### 2.1. Synthesis and Material Characterization

**Figure 2:**
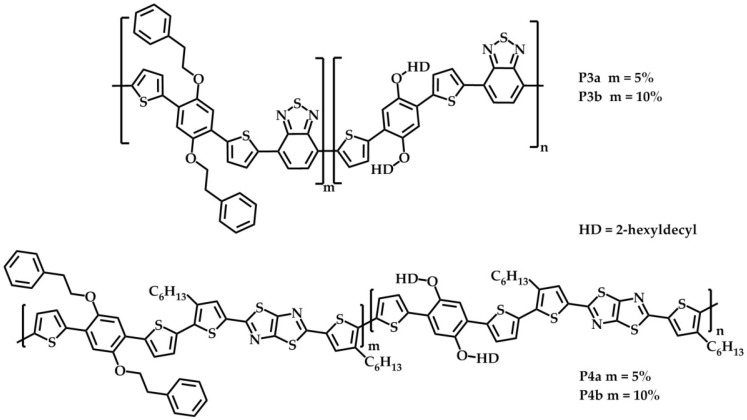
Chemical structures of the investigated statistical copolymers **P3a,b** and **P4a,b**.

In this study, six low bandgap copolymers were synthesized based on thiophene (T), phenylene (P), benzo[*c*][1,2,5]thiadiazole (BT) and thiazolo[5,4-*d*]thiazole (TzTz) building blocks (Figure 1 and Figure 2). **P1** and **P2**, bearing HD side chains on the P unit, were reported previously [12,29]. **P3a** and **P3b** were derived from **P1** and were prepared with partial substitution (5% and 10%, respectively [30]) of the HD by EtPh side chains. In the same way, **P4a** and **P4b** have the same backbone as **P2**, but also with partially-substituted side chains (5% and 10%, respectively).

**Scheme 1:**
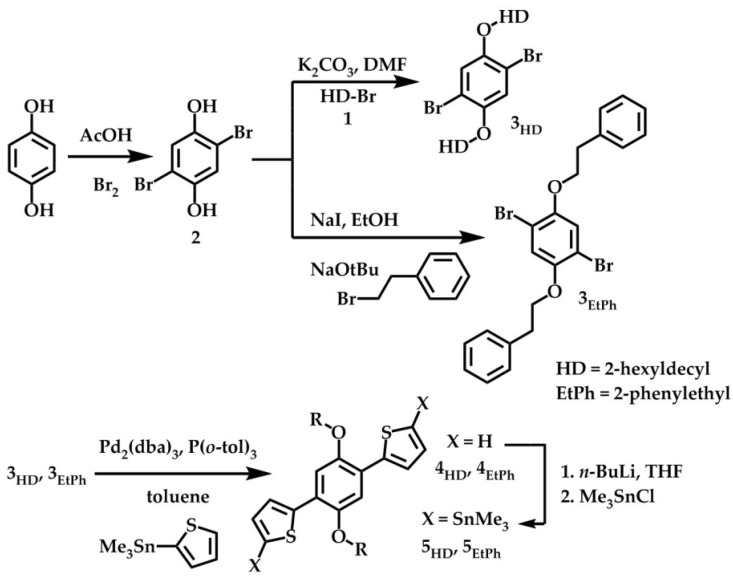
Synthetic routes for the monomers **5_HD_** and **5_EtPh_**.

The required distannylated monomers **5_HD_** and **5_EtPh_** for the envisaged Stille polycondensation were synthesized according to a procedure consisting of four steps starting from hydroquinone (Scheme 1) [16]. However, since the first two steps toward **5_EtPh_**, *i.e.*, bromination and alkylation of hydroquinone, resulted in a mixture of the di-alkylated (32%) and mono-alkylated (25%) product in rather low yields under the standard conditions used for the HD analogue, an alternative Williamson ether reaction was applied [26]. As such, 47% of the di-alkylated (and 29% of the mono-alkylated) product could be obtained. Thereafter, a Stille cross-coupling reaction with 2-(trimethylstannyl)thiophene and final stannylation of the obtained product afforded monomer **5_EtPh_**.

Standard Stille cross-coupling polymerization of monomers **5_HD_** and **5_EtPh_** with dibromo-BT yielded BT-based polymers **P1**, **P3a** and **P3b** (Figure 2). Whereas **P1** contains only one type of (HD) side chains, the synthesis of **P3a** and **P3b** was directed toward producing random copolymers with 5% and 10% of **5_EtPh_** units. Correspondingly, the dibrominated 2,5-dithienyl-TzTz monomer (synthesized using a literature procedure [31]) yielded polymer **P2** and copolymers **P4a** and **P4b** with 5% and 10% of **5_EtPh_**, respectively (Figure 2). Complete substitution of the HD side chains (100% of **5_EtPh_**) was only attempted for the BT-based backbone. However, the obtained polymer could not be analyzed due to a lack of solubility.

**Figure 3:**
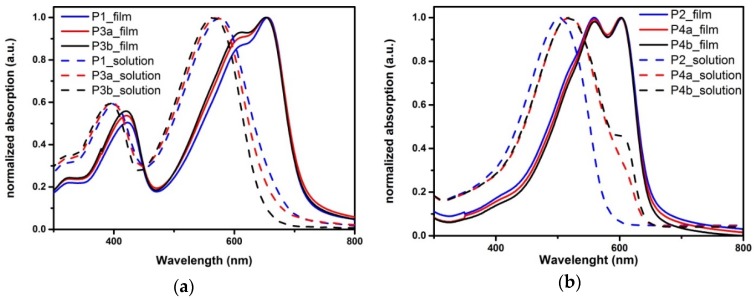
Normalized UV-VIS absorption spectra for the benzo[*c*][1,2,5]thiadiazole (BT)- (**a**) and thiazolo[5,4-*d*]thiazole (TzTz)- (**b**) based polymer series.

The synthesized polymers were characterized with standard techniques. ^1^H NMR spectra of the BT-based polymers were recorded in chloroform-*d_1_* (Figure S1a–c). No well-resolved spectra could be obtained for the TzTz-based polymers due to their low solubility in chloroform-*d_1_* and chlorobenzene-*d_5_*. The spectra of **P3a** and **P3b** showed two small signals at ~4.33 and 3.34 ppm for the two CH_2_ groups of the EtPh side chains. Integration of the signals at 3.34 ppm and the signals for the O–CH_2_ groups of the HD side chains at 4.08 ppm afforded monomer ratios of 4.9/95.0 for **P3a** and 9.4/90.0 for **P3b**, close to the monomer feed ratio. This shows that incorporation of the EtPh side chains was successful for both polymers. The molar masses of the polymers were determined by size exclusion chromatography (SEC), affording number-average molar masses (*M*_n_) of approximately 30–40 kDa (Table 1). In the UV-VIS spectra of the BT-based polymers in chloroform (CF) (Figure 3a), the absorption maximum (λ_max_), as well as the onset slightly shifted to lower wavelength with an increasing amount of EtPh substituents. In contrast, minor differences were seen in the UV-VIS spectra of the polymer films (Figure 3a). The optical bandgap of all BT-based polymers is 1.73 eV. The electrochemical bandgap, as determined by cyclic voltammetry (CV), decreased slightly upon elevating the amount of EtPh groups due to a minor increase of the highest occupied molecular orbital (HOMO) level (Table 1). On the other hand, the UV-VIS spectra of **P2**, **P4a** and **P4b** in chlorobenzene (CB) (Figure 3b) showed a shift toward a higher wavelength upon increasing the EtPh ratio, with additional formation of a shoulder at the low energy end. The absorption onset for the **P4** polymers was around 640 nm, whereas for **P2**, this appeared at 580 nm. The UV-VIS spectra of the polymer films, however, once again showed minor differences (Figure 3b). The optical bandgap for the three TzTz-based polymers is 1.91 eV. For this polymer series, there is also no significant difference in the electrochemical bandgap (values of ~2.22 eV).

### 2.2. Polymer Solar Cells

To investigate the influence of the partially-exchanged side chains on the photovoltaic performance and long-term PSC stability, both RC and SC bulk heterojunction solar cell devices were fabricated. The solar cell architecture for the RC devices consisted of PET/silver grid/PEDOT-PSS/ZnO/polymer:phenyl-C_61_-butyric acid methyl ester (PC_61_BM)/PEDOT-PSS/silver grid, with device areas of ~1 cm^2^ [12]. For the SC devices, the traditional device architecture glass/ITO/PEDOT-PSS/polymer:PC_61_BM/Ca/Al was applied, with an active device area of 3 mm^2^.

The photoactive layers of the RC devices prepared from **P1**, **P3a** and **P3b**, with a polymer to PC_61_BM ratio of 1:2 (wt %/wt %), were procured using *ortho*-dichlorobenzene (ODCB) as the processing solvent with a total concentration of 40 mg/mL, according to a previously-optimized procedure [12]. The coating was applied in air at 80 °C, and the photoactive layer thickness varied between 360, 450 and 540 nm. The PSCs were encapsulated between glass slides. As summarized in Table 2 and Figure S2a, **P1** granted a maximum PCE of 3.05% at an optimal layer thickness of 450 nm. For **P3a** and **P3b**, the highest PCEs were procured from active layer thicknesses of 360 nm, with performances of 2.65% and 2.17%, respectively. A slight decrease in the PCE could hence be observed upon increasing the amount of EtPh side chains, attributable to small reductions in open-circuit voltage (*Voc*) and mainly short-circuit current density (*Jsc*) (Table 2).

In a similar fashion, the photoactive layers for the SC devices based on **P1**, **P3a** and **P3b** were obtained from a 40-mg/mL solution in ODCB with a polymer to PC_61_BM ratio of 1:1.5 (wt %/wt %). In this way, PCEs of ~4% could be achieved (Table 2, Figure S2b). Once again, a small decrease in averaged PCE, *Voc* and *Jsc* could be observed upon increasing the amount of EtPh groups.

The PSCs made from the TzTz-based polymers showed less of a difference in the performance between the RC- and SC-processed solar cells (Table 2, Figure S2). The best results (maximum PCE of 2.73%) (Table 2, Figure S2a) for the RC-processed devices employing **P2** were achieved using a solvent mixture of ODCB and CB (4:1), a 1:1.5 (wt %/wt %; 40 mg/mL) ratio of **P2**:PC_61_BM and a layer thickness of 400 nm. These conditions were then also applied to **P4a** and **P4b** PSCs, yielding PCEs in the same range (2.72% and 2.76%, respectively).

The SC devices were also fabricated from ODCB:CB (4:1) as the processing solvent in a 1:1.5 (wt/wt%) ratio with a total concentration of 40 mg/mL for **P2** and **P4a** and 50 mg/mL for **P4b**. The elevated concentration for **P4b** was employed to warrant an optimal layer thickness. Upon insertion of the EtPh side chain moieties, a small increase in performance could be observed (Table 2, Figure S2b). The best device based on polymer **P4b** afforded a PCE of 3.21%. The *Voc* decreases within the series (which can tentatively be attributed to the lack of individual optimization). However, an improvement in *Jsc* can be seen by the partial exchange of the side chains (8.57 and 8.15 A/cm^2^ for **P4a** and **P4b**, respectively).

A general comparison of the different devices prepared shows, that the RC ones expectedly afforded lower efficiencies than the corresponding SC devices. This was, however, much more pronounced for the BT-based polymer series (1%–2%) as compared to the TzTz-based polymer series (0.1%–0.4%). The exchange of the side chain has a negative effect on the PCE of the BT-based PSCs, whereas the effect was neutral (RC) or even positive (SC) for the TzTz-based series.

The external quantum efficiency (EQE) spectra (Figure S3) for the BT-based RC PSCs showed a local maximum around 600 nm, with decreasing intensities for the different polymer solar cell devices with increasing amount of EtPh groups. On the other hand, there are no distinct differences in the curve progression and maximum EQE values (~37% at 520 nm) for the TzTz-based RC PSCs.

### 2.3. Stability Analysis

#### 2.3.1. Material Stability

The thermal properties of the polymers were analyzed by rapid-heat cool calorimetry (RHC) (Figure S4). RHC was chosen above regular differential scanning calorimetry (DSC) because of its increased sensitivity to thermal transitions as a result of the fast scanning rates and the low sample amounts required [32,33]. Nevertheless, it has barely been applied for low bandgap copolymers [19,25,28] to date. RHC analysis showed a semicrystalline character for the BT polymer group (Figure S4a). Whereas **P1** shows a sharp melting peak at 254 °C, partial exchange of the HD side chains with EtPh groups shifted the peak to higher temperatures (260 °C for **P3a** and 265 °C for **P3b**), but with similar melting enthalpy (11.5 J/g for **P3a** and **P3b** and 12.0 J/g for **P1**). The crystalline nature might be slightly suppressed due to the random incorporation of the thiophene-phenyl-thiophene units. While the melting peak temperature increases with an increased incorporation of the EtPh groups, the melting trajectory is broadened. A lower temperature shoulder seems more prominent for higher EtPh content. The Tg of these semicrystalline polymers could, however, not be detected in a reliable manner. In contrast, the thermograms for the TzTz polymer group show no melting peaks (in a temperature range of up to 300 °C), and these polymers seem completely amorphous. In this case, a Tg could be detected, increasing slightly between 148 and 156 °C with the amount of incorporated EtPh groups (Figure S4b). Both the melting peak temperature of the BT polymer group and the glass transition of the TzTz polymer group show a similar trend and are slightly increased with higher EtPh content.

**Figure 4:**
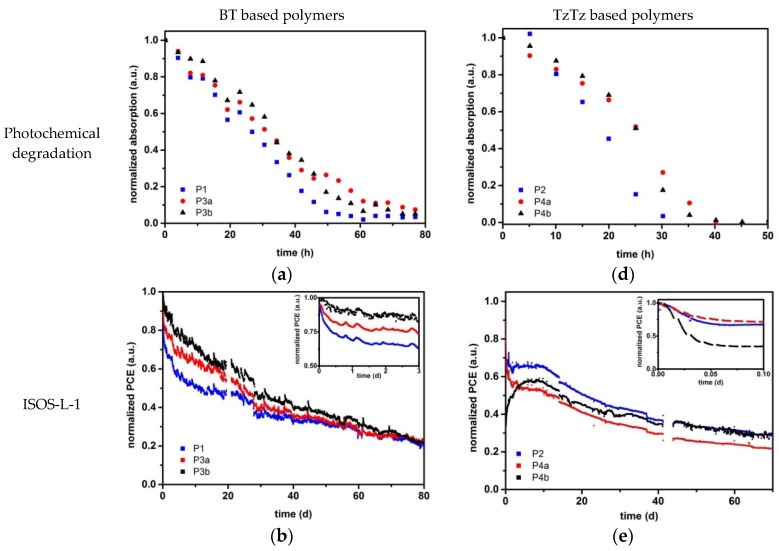

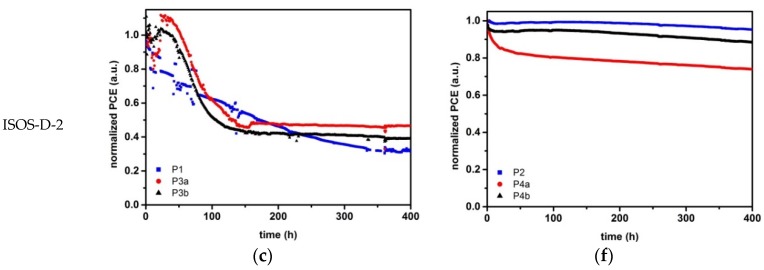
Maximum values of the normalized UV-VIS absorption of the polymer films as a function of time during sun irradiance (**a**,**d**), average lifetime measurements (ISOS-L-1 [35]) of the polymer solar cells (PSCs) normalized in terms of PCE (**b**,**e**) and the thermal degradation test (ISOS-D-2 [35]) of the PSCs normalized in terms of PCE (**c**,**f**) for the BT- (**a**–**c**) and TzTz- (**d**–**f**) based polymer series.

To investigate the photochemical stability of the pristine polymer materials, the UV-VIS absorption of the polymer films as a function of time was assessed at Air Mass (AM) 1.5 (1000 W/m^2^) conditions in an automatic set up (as described in the literature [34]). Upon prolonged exposure, the absorption of all polymers diminished strongly (with a slight blue shift; Figure S5), also clearly visible by a loss in film color during the experiment. From the normalized absorption degradation curves (Figure 4a,d), the degradation rates could be determined (see Table 1). The BT-based polymers exhibited degradation rates of approximately 1.66%/h, whereas the degradation rates for the TzTz-based polymers improved from 2.82%–2.13%/h with increasing amounts of EtPh side chains. The absorption of **P1**, **P3a** and **P3b** was almost completely gone after 60 h of light exposure. **P2** showed no absorption any more after 30 h, and **P4a** and **P4b** required 40 h of light exposure to show complete degradation. For **P2**, the photochemical stability improved in comparison to the previously reported **P2**-**12** (3.58%/h) [12]. Thus, manipulation of the side chain pattern to incorporate 10% of EtPh units does afford a further improved photochemical stability for the TzTz polymer series.

#### 2.3.2. Device Stability

For the RC-fabricated PSCs, the photochemical stability was investigated by a lifetime study under ISOS-L-1 standards [35] using at least three to five PSCs with layer thicknesses affording the best efficiencies. All cells were subdued to AM 1.5 (1000 W/m^2^) conditions for at least 70 days (1700 h). All RC devices were handled (coated, encapsulated) in the same way, such that a similar effect of UV light can be expected [36], and only relative values are reported. Initially, *IV* curves were measured every minute, with larger intervals of 10 and 30 min at longer timescales. The averages of the PV parameters over time were taken from the normalized values of each cell. Cell failures (when a cell broke down, but recovered to the initial value again) or measurement failures (when an error occurred in the system) were left out of the presented data. The results for the BT-based PSCs are shown in Figure 4b (PCE) and Figure S6a–c (*Voc*, *Jsc*, fill factor (FF)), whereas the averaged curves of the TzTz-based PSCs are shown in Figure 4e (PCE) and Figure S6d–f (*Voc*, *Jsc*, FF).

In the first phase (0–5 h) of the lifetime study, the PCE decreased quickly for all cells (Figure 4b,e), which is often referred to as the “burn-in” phase [35]. For the BT series, this burn-in was found to be more apparent for **P1** than for **P3a** and **P3b**. The EtPh side chains hence seem to have a beneficial influence on the initial degradation of the cells. As a general comparison point, the initial PCE was reduced to 80% of its initial value after 5, 29 and 111 h for **P1**, **P3a** and **P3b**, respectively. Afterwards, the PCE of all three PSCs decreased in a linear manner. However, the slope for **P3b** is larger than for **P3a** and **P1**. As such, the PCE of all BT-based devices decreased to ~25% of its initial value after 70 days. Even though the PSCs with a (higher) content of EtPh side chains are more stable in the burn-in phase, this effect was counteracted in the second phase of the lifetime testing. The *Voc*’s of **P1** and **P3a** have a similar stability, but the *Voc* of **P3b** started to degrade faster after ~20 days (Figure S6a). The change in *Jsc* is similar to the PCE decay. **P1** decreased faster in the burn-in phase, whereas **P3a** and **P3b** decreased at approximately the same rate (Figure S6b). The FF decreased in a similar manner for all three polymers (Figure S6c).

The lifetime behavior of the TzTz-based PSCs showed a different trend in the PV parameters (Figure 4e and Figure S6d–f). The efficiency of the solar cells incorporating polymers **P2** and **P4a** reduced rapidly to ~65% and ~70%, respectively, during the first 2 h (~0.1 day), whereas the performance of the devices based on **P4b** took a steep decay to ~35% during this rapid burn-in phase [35]. Hereafter, the PCE of the devices based on **P2** and **P4a** stayed rather constant until Day 10. In contrast, the PCE of the PSCs containing **P4b** remarkably recovered to ~60% again during this time. This constant process/recovery could be a result of slow photo-annealing [37] overlapping with the standard degradation. For **P2** and **P4b**, the increase in efficiency over the first 10 days seems to be higher than the decrease via degradation. After that, the PCEs of all three PSCs decreased continuously. However, the slope for **P4b** was somewhat smaller than for **P4a** and **P2**. As such, the PCE of **P4a** decreased to ~20%, whereas **P4b** and **P2** decreased to ~30% of their initial value after 70 days. For the PSCs based on TzTz**,** the influence of the EtPh side chains seems to be more effective in the second stage of the degradation. The behavior of the *Voc* was the same for all three PSC types, whereas the *Jsc* and FF developed similar to the PCE (but less distinct). In general, the disappearance of the absorption of the pure polymer films (Figure 4a,d and Figure S5), combined with the photochemical degradation of the corresponding solar cells (toward similar relative values for all polymers; Figure 4b,e), seems to point to a common photo-degradation pathway for both material series. Rivaton *et al.* have proposed a photochemical degradation route in which the alkoxy side chains on the phenyl building block in MDMO-PPV are cleaved off via photolysis [38]. Since the degradation profiles observed here look very similar and the same type of alkoxy side chains are employed, we can assume a similar behavior for this polymer series, practically independent of the presence of the phenyl end groups.

The thermal stability of the OPV devices was also investigated, more specifically for the SC PSCs in a nitrogen-containing glovebox at 85 °C using a dedicated degradation chamber according to the ISOS-D-2 protocol [35]. For the solar cells employing **P3a** and **P3b**, an initial (thermal annealing) phase in which the PCE increased slightly could be observed, followed by a rapid decrease to ~50% and ~40% of the initial value, respectively (Figure 4c). Afterwards, the performance of these two materials stayed constant for the next 250 h. In comparison, the degradation of the devices based on **P1** occurred in a more linear manner. Similar to the two analogous statistical copolymers, the degradation levels out after a certain time (350 h for **P1**), at which ~30% of the initial PCE was retained (Figure 4c). The *Jsc* followed a similar trend as the PCE, whereas the *Voc* remained rather constant (Figure S7). These studies clearly show that the incorporation of EtPh side chains has a positive effect on the stability of the PSCs based on the BT polymer series in the beginning of the tests. However, for a longer term, it is clear that other degradation processes counteract this.

For the devices based on TzTz copolymers **P4a** and **P4b**, an initial burn-in phase was observed in which the PCE decreased to 85 and 95%, respectively, followed by a slow linear decay of up to another 10% over 350 h. In contrast, PSCs based on **P2** showed a remarkably high thermal stability over the entire time scale, with a retained efficiency of up to 95% of the initial value after 400 h of thermal stress (Figure 4f). The drop in *Jsc* (Figure S7e) of **P2** is balanced out by the rise in FF (Figure S7f). It must be noted, however, that the *Jsc* drop is more present for **P2** than for **P4b** (Figure S7e), but this is not reflected in the PCE due to the rise in FF for **P2**.

**Figure 5:**
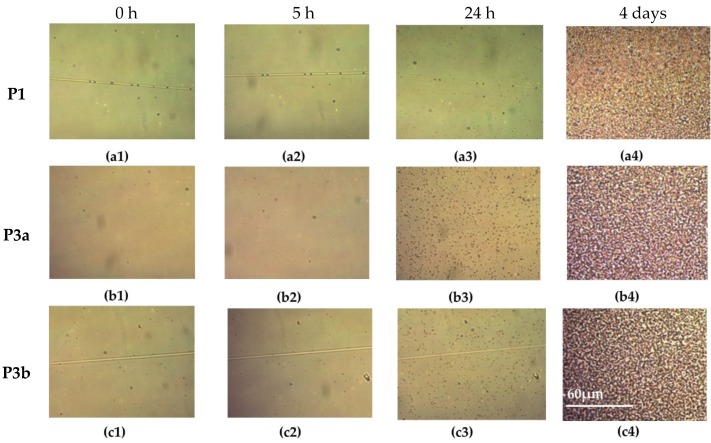
Optical microscope images (50× magnification) of the active layers of RC PSCs based on **P1** (**a**), **P3a** (**b**) and **P3b** (**c**) after exposure to 85 °C for 0 h (1), 5 h (2), 24 h (3) and four days (4).

**Figure 6:**
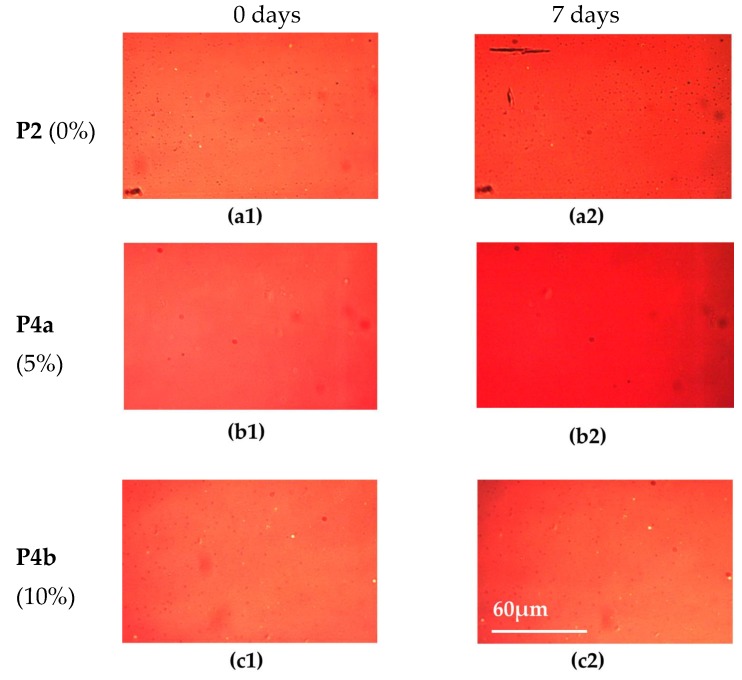
Optical microscope images (with 50× magnification) of the active layers of RC PSCs based on **P2** (**a**), **P4a** (**b**) and **P4b** (**c**) after exposure to 85 °C for zero days (1) and seven days (2).

To gain more insight into the effect of the exchanged side chains on the thermal stability, non-encapsulated RC PSCs were heated at 85 °C for four and seven days, respectively, and optical microscope images were taken at different time intervals (Figure 5 and Figure 6). For the BT-based PSCs, the images show no clear differences or distinct features within the first 5 h of heating. After 24 h, however, the formation of particles in the photoactive layer becomes apparent, and after four days, all films show a rather strong phase separation, which can be connected with the degradation during the long time exposure to thermal stress (Figure 4c). To further assess the thermal stability, in particular with respect to the changes in the morphology of the photoactive layer, transmission electron microscopy (TEM) measurements were performed on the active layers of **P1**, **P3a** and **P3b** taken from the SC PSCs before and after the application of the thermal stress of 85 °C for 400 h (Figure S8). Initially, the photoactive layer blends showed no remarkable features. After the application of the thermal stress, however, large phase-separated domains were formed. Unfortunately, this could not directly be related to *T*_g_ (as these could not be observed for the BT polymer series). The formation of fullerene crystals is most likely responsible for the loss in *Jsc* (and PCE), as observed before [39].

The optical microscope images taken from the photoactive layer blends employing **P2**, **P4a** and **P4b** (Figure 6) show no particular features after inducing a thermal stress of 85 °C for seven days. This result is in good correlation with the minor efficiency loss during the thermal stress test on the SC PSCs (Figure 4f). As expected, since the *T*_g_’s of these polymers are well above the degradation temperature (85 °C), the blend components remain intermixed (no phase separation), resulting in a higher thermal stability across this series. Because of the minor rise in *T*_g_ upon implementation of the low ratio of EtPh side chains, no noticeable difference can be seen within the series (in contrast to the previous work in which 100% of the dimethyloctyloxy side chains of MDMO-PPV were exchanged) [26].

Summarizing, the ISOS-L-1 lifetime study of the RC devices showed an improved stability for the PSCs based on the BT polymers with EtPh groups within the first 20 days of the study, whereas the devices based on the EtPh-decorated TzTz-based polymers had an improved stability during the last 50 days of the study. The ISOS-D-2 thermal degradation study of the SC devices employing the BT-based polymers with EtPh groups showed a rapid decrease in solar cell performance during the first part of the study, followed by a stable PCE at ~40% of the initial value, whereas the PCE of P1 continued decreasing. For the TzTz-based polymer series, the PSCs based on P2 showed a small loss (up to 5%) of the initial PCE, whereas the decrease of the PSCs based on the EtPh derivatives was higher (up to 25% for **P4a**). The comparison between the two different polymer backbones makes it clear that the exchange of some of the HD side chains by EtPh groups has a notably different influence on the PV parameters of the resulting PSCs. The lifetime measurements showed opposite effects for the two different polymer backbones with different HD/EtPh ratios. In general, the differences are quite remarkable for a maximum of 10% exchanged side chains. Unfortunately, no general trends, clear design rules nor significantly improved stabilities were realized at this stage, in contrast to previous work. Nevertheless, the results do show that side chain engineering is a powerful approach to optimize PSC efficiency, as well as stability, and provide the impetus for further research efforts.

## 3. Experimental Section

### 3.1. Material and Methods

Unless stated otherwise, all reagents and solvents were obtained from Sigma-Aldrich (St. Louis, MO, USA) and used without further purification. PC_61_BM (phenyl-C_61_-butyric acid methyl ester) (PV-A600) was purchased from Merck Chemicals (RC) and Sollenne (SC), while PEDOT:PSS (poly(3,4-ethylenedioxythiophene):poly(styrenesulfonic acid)) (Orgacon EL-P 5010) was obtained from Agfa (RC) or Heraeus Clevios (SC). Thermally-curable Ag (PV-410) was received from DuPont, and UV-curable adhesive (DELO, Katiobond LP 655) was from DELO. Ca and Al were acquired from Alpha Aesar and Kurt J. Lesker, respectively.

NMR spectra were recorded in CDCl_3_ on a 500-MHz Bruker spectrometer (Bruker Corporation, Billerica, MA, USA) and a 300-MHz Varian Inova-300 spectrometer (Varian Inc., Palo Alto, CA, USA).

Analytical size exclusion chromatography (SEC) was performed using a Spectra Series P100 (Agilent, Santa Clara, UT, USA) pump equipped with two mixed-B columns (10 μm, 2 cm × 30 cm, Polymer Laboratories, Varian Inc., Palo Alto, CA, USA) and an Agilent 1100 DAD UV detector (Agilent, Santa Clara, UT, USA). Chlorobenzene was used as the eluent at a flow rate of 1.0 mL·min^−1^ and a temperature of 60 °C. Molar masses were determined with UV detection at λ_max_ of the polymer and relative to polystyrene standards.

UV-VIS spectra were recorded on a UV-3600 spectrophotometer (Schimadzu Scientific Instruments, Columbia, PA, USA) or Cary 5000 UV-VIS-NIR spectrophotometer (Agilent Technologies, Victoria, Australia). The spectra were obtained from chloroform (BT-based polymers) or chlorobenzene (TzTz-based polymers) solutions and spin-coated films on glass or quartz slides. Films were spin-coated from solutions with a concentration of ~10 mg/mL. Diluted solutions (~0.01 mg/mL) were used for the solution UV-VIS measurements.

Electrochemical measurements were performed with an Autolab PGSTAT 30 potentiostat/galvanostat (Eco Chemie, Utrecht, The Netherlands) using a three-electrode microcell with a platinum working electrode, a platinum counter electrode and an Ag/AgNO_3_ reference electrode (silver wire dipped in a solution of 0.01 M AgNO_3_ and 0.1 M NBu_4_PF_6_ in anhydrous MeCN). The reference electrode was calibrated against ferrocene/ferrocenium. Anhydrous MeCN containing 0.1 M NBu_4_PF_6_ was used as the electrolyte and was degassed with argon prior to each measurement. To prevent air from entering the system, the experiments were carried out under a curtain of argon. The polymer samples were dissolved in chloroform. The working electrode was dipped into the polymer solution and dried at room temperature before the measurement. Cyclic voltammograms were recorded at a scan rate of 100 mV·s^−1^. The HOMO-LUMO energy levels of the products were estimated using the obtained CV data. For the conversion of V to eV, the onset potentials of the first oxidation/reduction peaks were used and referenced to ferrocene/ferrocenium, which has an ionization potential of −4.98 eV *vs*. vacuum. This correction factor is based on a value of 0.31 eV for Fc/Fc^+^ *vs.* SCE [40] and a value of 4.68 eV for SCE *vs.* vacuum [41]: $E_{\text{HOMO}/\text{LUMO}}\left(\text{eV}\right)=-4.98-E_{\text{onset ox}/\text{red}}^{\text{Ag}/\text{AgNO}3}\;\left(V\right)\;+-E_{\text{onset ox}/\text{red}}^{\text{Ag}/\text{AgNO}3}\;\left(V\right)\;$. The accuracy of measuring redox potentials by CV is about 0.01–0.02 V. Reproducibility can be less, because the potentials do depend on concentration and temperature.

Differential scanning calorimetry (DSC) was performed on a Rapid-Heating-Cooling (RHC) instrument (TA Instruments, New Castle, PA, USA) using a liquid nitrogen cooling system and purging with helium (12 mL·min^−1^). The RHC cell is heated by four quartz halogen lamps. The samples (~0.25 mg) were enclosed in dedicated aluminum crucibles and lids.

The photochemical stability of the polymer films prepared by SC (for the UV-VIS measurements) was analyzed using a dedicated setup described before [34].

The PSC area was studied using a light beam induced current (LBIC) technique described in the literature [42].

An Axioskop from Zeiss (Oberkochen, Germany) was used for optical microscopy.

The external quantum efficiency (EQE) measurements were carried out using a system from PV Measurements, Inc. (Boulders, CO, USA) at 10-nm intervals between 300 and 800 nm. A calibrated Si cell was used before the experiment as a reference.

Transmission electron microscopy (TEM) measurements were performed on a FEI Tecnai Spirit (Eindhoven, The Netherlands)using an accelerating voltage of 120 kV. TEM samples were prepared from pristine devices or from the devices utilized in the automated degradation chamber. By washing away the PEDOT:PSS layer with water, freestanding films were obtained.

ISOS-L-1 lifetime tests were performed using a solar simulator with AM 1.5 (1000 W/m^2^) conditions. The IV-curve tracing of the PSCs was performed using an automated acquisition setup with a Keithley 2400 SMU (Keithley Instruments, Eindhoven, The Netherlands).

ISOS-D-2 degradation tests were performed on solar cells positioned in an automated degradation chamber in nitrogen atmosphere (glovebox) with a constant temperature of 85 °C. During an initial phase, the *IV*-characteristics were measured every 30 min and at a later stage every hour, using a White 5500 K LED (LED Engin, San José, USA) from which a maximum illumination intensity of 0.53 sun could be obtained.

### 3.2. Monomer Synthesis

2-Hexyldecylbromide (**1**), 2,5-dibromobenzene-1,4-diol (**2**), 1,4-dibromo-2,5-bis(2-hexyldecyl oxy)benzene (**3_HD_**), 2,2′-[2,5-bis(2-hexyldecyloxy)-1,4-phenylene]dithiophene (**4_HD_**), {[2,5-bis(2-hexyldecyloxy)-1,4-phenylene]bis(thiophene-5,2-diyl)}bis(trimethylstannane) (**5_HD_**) and 2,5-bis(5-bromo-4-hexylthiophene-2-yl)thiazolo[5,4-*d*]thiazole were synthesized according to various literature procedures [14,16,29,31].

#### 3.2.1. {[(2,5-Dibromo-1,4-phenylene)bis(oxy)]bis(ethane-2,1-diyl)}dibenzene (**3_EtPh_**)

**3_EtPh_** was synthesized using a similar reaction as reported in the literature [26]. Compound **2** (10.0 g, 37.3 mmol) and potassium tert-butanolate (10.0 g, 89.5 mmol) were dissolved in absolute ethanol (20 mL), and the solution was degassed for 20 min. First, 2-phenylethyl bromide (11.5 mL, 82.1 mmol) was added dropwise to this solution and then sodium iodide (0.31 g, 2.1 mmol) all at once, followed by heating under reflux overnight. After cooling down, the organic solvent was removed, and water was added to dissolve the inorganic salts. The water phase was extracted three times with dichloromethane, followed by drying with MgSO_4_, filtration and removal of the organic solvent under reduced pressure. In the end, 8.42 g of **3_EtPh_** were obtained as a white powder by recrystallization from ethanol (yield: 47%). ^1^H NMR (CDCl_3_, 500 MHz) δ: 3.13 (*t*, *J* = 7.0 Hz, 4H), 4.83 (*t*, *J* = 7.0 Hz, 4H), 7.06 (s, 2H), 7.32–7.34 (m 10H). ^13^C NMR (CDCl_3_, 125 MHz) δ: 35.9, 71.1, 111.3, 118.6, 126.8, 128.6, 129.3, 138.0, 150.1.

#### 3.2.2. 2,2′-(2,5-Diphenethoxy-1,4-phenylene)dithiophene (**4_EtPh_**)

A general Stille cross-coupling reaction was performed according to the procedure reported for **4_HD_** [29]. Recrystallization from chloroform and methanol yielded a yellow solid (yield: 74%). ^1^H NMR (CDCl_3_, 500 MHz) δ: 3.23 (*t*, *J* = 7.2 Hz, 4H), 4.30 (*t*, *J* = 7.2 Hz, 4H), 7.06 (dd, *J* = 5.1, 3.7 Hz, 2H), 7.22 (s, 2H), 7.32–7.33 (m, 12H), 7.43 (dd, *J* = 3.7, 1.2 Hz, 2H). ^13^C NMR (CDCl_3_, 125 MHz) δ: 36.1, 70.8, 113.5, 123.4, 125.7, 125.8, 126.7, 127.0, 128.7, 129.2, 138.3, 139.1, 149.4.

#### 3.2.3. [(2,5-Diphenethoxy-1,4-phenylene)bis(thiophene-5,2-diyl)]bis(trimethylstannane) (**5_EtPh_**)

A general method was applied for the distannylation of **4_EtPh_** [16]. A yellow solid was obtained, which was washed three times with methanol (yield: 73%). ^1^H NMR (CDCl_3_, 500 MHz) δ: 0.41 (s, 12H), 3.23 (*t*, *J* = 7.2 Hz, 4H), 4.31 (*t*, *J* = 7.2 Hz, 4H), 7.15 (d, *J* = 3.5 Hz, 2H), 7.22 (s, 2H), 7.33–7.34 (m, 10H), 7.56 (d, *J* = 3.5 Hz, 2H). ^13^C NMR (CDCl_3_, 125 MHz) δ: −8.1, 36.2, 70.6, 113.6, 123.3, 126.7, 127.1, 128.7, 129.2, 129.3, 135.4, 138.1, 138.4, 145.2, 149.2.

### 3.3. Polymer Synthesis

#### 3.3.1. General Procedure for the Stille Cross-Coupling Polymerization

4,7-Dibromobenzo[c][1,2,5]thiadiazole or 2,5-bis(5-bromo-4-hexylthiophene-2-yl)thiazolo[5,4-*d*]thiazole were polymerized in a standard procedure with different ratios of **5_HD_** and **5_EtPh_** (0/5%/10%), resulting in polymers **P1**, **P2**, **P3a**, **P3b**, **P4a** and **P4b**. The monomers were dissolved in anhydrous toluene (70 mg monomer/mL). Then, 0.03 equivalents (eq.) of tris(dibenzylideneacetone)dipalladium(0) and 0.09–0.18 eq of tris(o-tolyl)phosphine were added before the mixture was refluxed for at least 20 h. The raw polymer solution was worked up by the precipitation in methanol. The solids were filtrated, and Soxhlet extraction was performed with methanol and n-hexane. The leftover polymers were dissolved in hot CF and precipitated into methanol, filtrated and dried under vacuum. The polymers were characterized by ^1^H NMR, SEC and UV-VIS.

#### 3.3.2. Poly{[2,5-bis(2-hexyldecyloxy)phenylene]-*alt*-[4,7-di(thiophene-2-yl)benzo[*c*][1,2,5]thiadiazole]} (**P1**)

Blue solid. 96% yield. Mn = 42 kDa, polydispersity index (PDI) = 1.9. ^1^H NMR (CDCl_3_, 300 MHz) δ: 0.85–0.97 (m, 12H), 1.25–1.54 (m, 44H), 1.70 (br, 4H), 2.01 (br, 2H), 4.08 (br, 4H), 7.10–8.19 (m, 8H).

#### 3.3.3. Poly{2,2′-[5,5′-(2,5-bis(2-hexyldecyloxy)-1,4-phenylene)dithiophene]-*alt*-[2,5-bis(4-hexyl thiophen-2-yl)thiazolo[5,4-*d*]thiazole]} (**P2**)

Blue solid. 79% yield. Mn = 44 kDa, PDI = 1.9.

#### 3.3.4. BT-Based Statistical Copolymers

**P3a**: Blue solid. 98% yield. Mn = 39 kDa, PDI = 2.1. ^1^H NMR (CDCl_3_, 300 MHz) δ: 0.85–0.97 (m, 12H), 1.25–1.54 (m, 44H), 1.75 (br, 4H), 2.01 (br, 2H), 3.35 (br, 5% 4H), 4.08 (br, 4H), 4.33 (br, 5% 4H), 7.10–8.18 (m, 8H).

**P3b**: Blue solid. 97% yield. Mn = 43 kDa, PDI = 2.1. ^1^H NMR (CDCl_3_, 300 MHz) δ: 0.85–0.97 (m, 12H), 1.25–1.54 (m, 44H), 1.70 (br, 4H), 2.01 (br, 2H), 3.34 (br, 10% 4H), 4.05 (br, 4H), 4.33 (br, 10% 4H), 7.10–8.19 (m, 8H).

#### 3.3.5. TzTz-Based Statistical Copolymers

**P4a**. Blue solid. 95% yield. Mn = 30 kDa, PDI = 3.1.

**P4b**. Blue solid. 89% yield. Mn = 78 kDa, PDI = 1.5.

### 3.4. Device Preparation and Testing

The RC PSCs, with a general structure PET/silver grid/PEDOT-PSS/ZnO/polymer:PC_61_BM/PEDOT-PSS/silver grid, were produced on Flextrode substrates (PET/Silver grid/PEDOT-PSS/ZnO) [43,44] using a laboratory roll coater at ambient conditions [45,46].

The active layers based on **P1**, **P3a** and **P3b** were coated at 80 °C with a total concentration of 40 mg/mL with a polymer:PC_61_BM ratio of 1:2 (wt/wt%) using ODCB as the processing solvent [12]. The thickness of the active layer was varied (360, 450 and 540 nm). The photoactive layers based on **P2**, **P4a** and **P4b** were coated from a solvent mixture ODCB:CB (4:1) with a polymer to PC_61_BM ratio of 1:1.5 (wt %/wt %) and a layer thickness of 400 nm at 80 °C. Approximately five PSCs per coating batch were encapsulated between two glass slides using UV-curable adhesive. The adhesive was activated for a few minutes using a solar simulator. The exact area (~1 cm^2^) of all cells was determined using LBIC [29]. The RC PSCs were tested under AM 1.5 (1000 W/m^2^) conditions. *IV*-curves were recorded to determine the PV parameters (open-circuit voltage (*Voc*), short-circuit current density (*Jsc*), fill factor (FF) and PCE). For the RC thermal stability tests, two non-encapsulated RC PSCs were heated on a hot plate at 85 °C, and the active layer morphology was probed at regular intervals using an optical microscope.

For the spin-coated devices, the traditional solar cell architecture consisting of glass/ITO/PEDOT:PSS/polymer:PC_61_BM/Ca/Al was employed. Prior to processing, the indium tin oxide (ITO, Kintec, 100 nm, 20 Ohm/sq) -coated glass substrates were thoroughly cleaned using soap, demineralized water, acetone, isopropanol and a UV/O_3_ treatment. PEDOT:PSS was subsequently deposited at a thickness of ~30 nm, and annealing at 130 °C for 15 min was applied to remove any residual water. Further processing was carried out in a nitrogen-containing glovebox (O_2_/H_2_O < 1 ppm), starting with the deposition of the photoactive layer by spin-coating. For **P1**, **P3a** and **P3b**, blend solutions with a total concentration of 40 mg/mL with a polymer:PC_61_BM ratio of 1:1.5 (wt %/wt %) were prepared using ODCB as the processing solvent. For **P2**, **P4a** and **P4b**, the processing solvent was switched to ODCB:CB (4:1). The polymer:PC_61_BM ratio was retained at 1:1.5 (wt %/wt %), similar to the BT series, and total concentrations of 40 mg/mL for **P2** and **P4a** and 50 mg/mL for **P4b** were employed. Finally, the devices were finished off by vacuum deposition of the top electrodes, Ca and Al, with layer thicknesses of 30 and 80 nm, respectively. The SC device performance measurements were done using a Newport Class A solar simulator (Model 91195A), calibrated with a silicon solar cell to give an AM 1.5 spectrum.

## 4. Conclusions

Four new donor-acceptor-type low bandgap copolymers were successfully synthesized by manipulation of the side chains of two copolymers (**P1** and **P2**) based on benzo[*c*][1,2,5]thiadiazole (BT) and thiazolo[5,4-*d*]thiazole (TzTz) acceptor units, respectively. The initial goal of this work was to elevate the *T*_g_ of these two polymer series upon exchanging a small amount (5%–10%) of 2-hexyldecyloxy by 2-phenylethoxy (EtPh) side chains and thereby increasing the (thermal) stability of the resulting PSCs, as successfully demonstrated in past research efforts [26,27,28]. As such, partial exchange (5% or 10%) of the solubilizing 2-hexyldecyloxy side chains on the bis(thienyl)dialkoxybenzene donor parts by EtPh substituents was successfully performed. Unfortunately, due to the (semi)crystalline nature of the BT-based polymers, no distinct *T*_g_ could be observed, and given the small amount of functional moieties, the *T*_g_ of the TzTz-based polymer series only elevated slightly. The polymers were applied in polymer solar cells (PSCs) via roll-coating (RC) and spin-coating (SC) protocols. The power conversion efficiencies (PCEs) of the small-scale SC devices were higher than those for the RC devices for both polymer series, although the difference was noticeably smaller for the TzTz series. The BT-based RC devices showed a reduction in PCE with increasing amount of EtPh groups (3.05% down to 2.17%), whereas the devices based on TzTz had similar PCE values (~2.7%) for the entire series. In comparison, the SC devices showed a small (average) PCE reduction with increasing amount of EtPh side chains for both polymer groups.

The photochemical degradation rates for the BT-based polymers were similar (~1.6%/h), whereas the rate for the TzTz-based polymers decreased from 2.8 down to 2.1%/h with an increasing amount of EtPh groups. The lifetime study (up to 80 days) of the RC devices under constant sun irradiation demonstrated an improved stability in the first phase of the study (up to 20 days) for the BT-based PSCs containing a higher amount of EtPh side chains, whereas for the TzTz-based devices, the stability improved in the last stage of the study (after 20 days). Since both polymer series exhibited a similar behavior under illumination (discoloration of the films, comparable residual relative PCEs after degradation, *etc*.), it is likely that these polymers follow a similar degradation pathway in which the alkoxy side chains are cleaved off via a photolysis mechanism [37], thereby making obsolete any influence of the side chain manipulation.

Thermal stability studies (400 h at 85 °C) of the SC devices using the BT-based polymers with manipulated side chains (**P3a** and **P3b**) showed a rapid loss in the first stage, followed by a stable regime (of ~40% of the initial PCE), whereas the PCE of the reference polymer **P1** continued decreasing. PSC devices prepared from the TzTz-based reference polymer **P2** showed a minor loss (5% of the initial PCE) during the thermal stress study, whereas the loss for the PSCs based on the polymers with manipulated side chains (**P4a** and **P4b**) was higher (up to 25% for **P4a**). For the (semicrystalline) BT-based polymer series, phase separation and fullerene crystallization were identified as the main malefactors resulting in a loss in *Jsc*, and hence, PCE. For the TzTz polymers, distinct *T*_g_’s well above the degradation temperature (85 °C) were determined, thereby reducing the phase separation tendency upon applying thermal stress. Due to the low EtPh incorporation ratio, only a slight elevation of the *T*_g_ could be achieved, limiting the effect on the thermal stability.

In conclusion, the application of altered side chain patterns on different polymer backbones, as a potential route to enhance solar cell stability, demonstrated varying results on PSC performance, as well as on the lifetime and thermal stability. However, no conclusive positive results were achieved at this stage. It hence remains difficult to generalize observations made for specific conjugated polymer derivatives (and the resulting devices) toward the whole range of materials currently investigated in the field. Future work will encompass further studies on different types and ratios of side chains and their effect on different polymer backbones in our pursuit to find a correlation between side chain structure, polymer backbone structure and device stability. Degradation of the organic/metal electrode interfaces will be investigated in more detail, as well.

## Figures and Tables

**Table 1:** Molecular, optical and electrochemical properties of the synthesized polymers.

Polymer	EtPh ^1^	*M*_n_^2^	PDI ^2^	λ_onset_^3^ Solution	λ_onset_^3^ Film	Δ*E*_op_^3^	Δ*E*_ec_^4^	HOMO ^4^	LUMO ^4^	*R*_d_^5^
P1	0%	42	1.9	669	713	1.73	2.07	−5.37	−3.30	1.66
P3a	5%	39	2.1	656	715	1.73	2.03	−5.34	−3.31	1.63
P3b	10%	43	2.1	645	717	1.73	1.99	−5.31	−3.32	1.69
P2	0%	44	1.9	580	650	1.91	2.24	−5.24	−3.01	2.82
P4a	5%	30	3.1	640	647	1.91	2.21	−5.20	−2.99	2.60
P4b	10%	78	1.5	640	646	1.91	2.22	−5.21	−2.99	2.13

^1^ Percentage of EtPh side chains in the polymer; ^2^ number-average molar mass (in kDa) and polydispersity index (PDI) determined by size exclusion chromatography in chlorobenzene at 60 °C against polystyrene standards; ^3^ in nm (λ_onset_) and in eV (Δ*E*_op_) (determined by UV-VIS spectroscopy); ^4^ in eV (determined by cyclic voltammetry); ^5^ *R*_d_ = degradation rate (%/h) calculated from photochemical stability measurements of the pure polymer films.

**Table 2:** Current-voltage (*IV)* parameters ^1^ (*Voc*, *Jsc*, fill factor (FF) and power conversion efficiency (PCE)) for the polymer:phenyl-C_61_-butyric acid methyl ester (PC_61_BM) RC and SC solar cells based on **P1**, **P2**, **P3a**, **P3b**, **P4a** and **P4b**.

Polymer	Method ^2^	*Voc* (V)	*Jsc* (mA/cm^2^)	FF	PCE (Best) (%)
**P1**	RC ^3^	0.75	7.83	0.47	2.81 (3.05)
	SC ^4^	0.74	10.38	0.56	4.31 (4.42)
**P3a**	RC ^3^	0.74	6.52	0.52	2.51 (2.65)
	SC ^4^	0.72	10.22	0.56	4.15 (4.54)
**P3b**	RC ^3^	0.73	5.76	0.50	2.10 (2.17)
	SC ^4^	0.71	9.41	0.55	3.68 (4.09)
**P2**	RC ^5^	0.69	6.69	0.61	2.67 (2.73)
	SC ^6^	0.72	6.60	0.58	2.76 (2.92)
**P4a**	RC ^5^	0.68	6.95	0.59	2.59 (2.72)
	SC ^6^	0.65	8.57	0.55	3.06 (3.06)
**P4b**	RC ^5^	0.69	7.00	0.60	2.66 (2.76)
	SC ^6^	0.61	8.15	0.63	3.13 (3.21)

^1^ *IV* parameters are averaged over 5 devices for RC and 2–8 devices for SC; ^2^ RC: roll-coating in air with an inverted device geometry of ~1 cm^2^ (PET substrate/Ag grid/PEDOT- PSS/ZnO/polymer:PC_61_BM/PEDOT-PSS/Ag grid); SC: spin-coating under inert atmosphere with a device geometry of 3 mm^2^ (glass/ITO/PEDOT-PSS/polymer:PC_61_BM/Ca/Al); ^3^ RC of polymer:PC_61_BM blends in a 1:2 ratio processed from *ortho*-dichlorobenzene (ODCB), with a layer thickness of 450 nm for **P1** and 360 nm for **P3a** and **P3b**; ^4^ SC of polymer:PC_61_BM blends in a 1:1.5 ratio processed from ODCB; ^5^ RC of polymer:PC_61_BM blends in a 1:1.5 ratio processed from ODCB/CB = 4/1, with a layer thickness of 400 nm; ^6^ SC of polymer:PC_61_BM blends in a 1:1.5 ratio processed from ODCB/CB = 4/1.

## Supplementary Materials

The following are available online at www.mdpi.com/1996-1944/9/3/181/s1. Figure S1a, ^1^H NMR spectrum of polymer **P1** in CDCl_3_, Figure S1b: ^1^H NMR spectrum of polymer **P3a** (5% EtPh) in CDCl_3_, Figure S1c: ^1^H NMR spectrum of polymer **P3b** (10% EtPh) in CDCl_3_, Figure S2: *IV*-curves for the best PSCs made via RC and SC using the BT- and TzTz-based polymer series, Figure S3: External quantum efficiency (EQE) spectra for the PSCs made via RC using the BT- and TzTz-based polymer series, Figure S4: Rapid-heat cool calorimetry (RHC) thermograms for the BT- and the TzTz-based polymers, Figure S5: Evolution of the UV-VIS absorption profiles during the photochemical stability tests, Figure S6: Average lifetime measurements for the RC devices using the BT- and TzTz-based polymer series showing the normalized *Voc*, *Jsc* and FF trends, Figure S7: Thermal degradation tests of the SC devices using the BT- and TzTz-based polymer series showing the normalized *Voc*, *Jsc* and FF trends, Figure S8: TEM images of the active layers of SC PSCs based on **P1**, **P3a** and **P3b** before and after exposure to 85 °C for 400 h.
