# Supplementary material for: The Influence of Conjugated Polymer Side Chain Manipulation on the Efficiency and Stability of Polymer Solar Cells
Source: Materials (Basel). 2016 Mar 9;9(3):181. doi: 10.3390/ma9030181 (PMC5456685; doi:10.3390/ma9030181)
Supplement: Supplementary file 1 [file materials-09-00181-s001.pdf]

Supplementary Materials: The Influence of Conjugated Polymer Side Chain Manipulation on the Efficiency and Stability of Polymer Solar Cells

Ilona M. Heckler, Jurgen Kesters, Maxime Defour, Morten V. Madsen, Huguette Penxten, Jan D'Haen, Bruno Van Mele, Wouter Maes and Eva Bundgaard

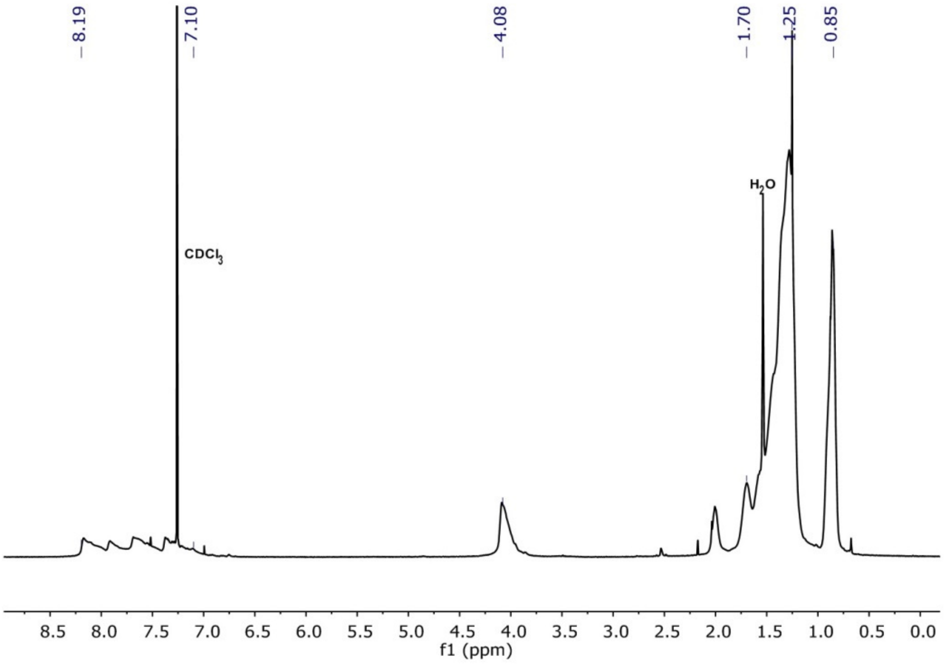

(**a**)

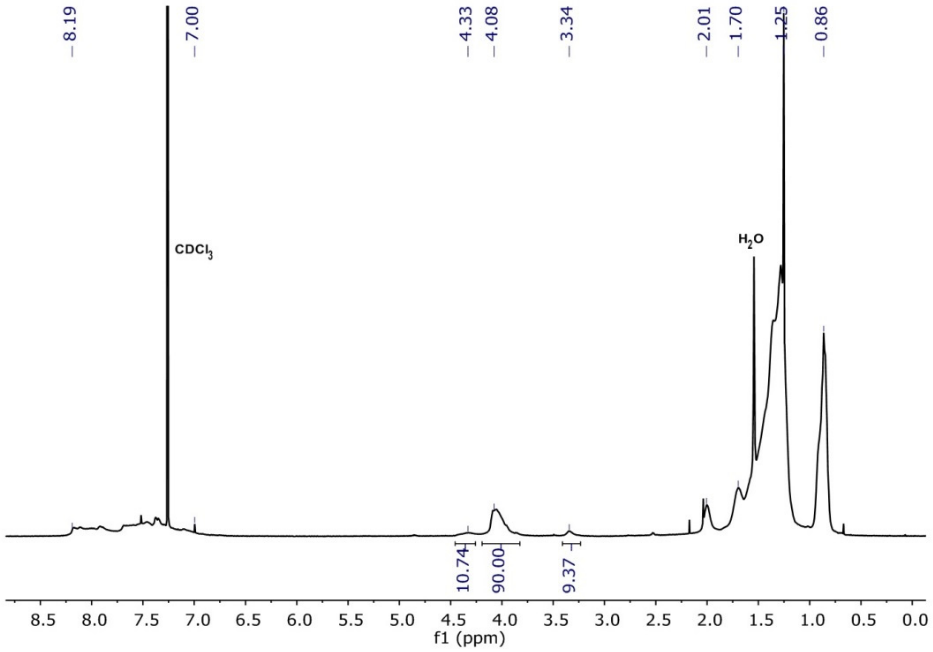

(**b**)

Figure S1. *Cont.*

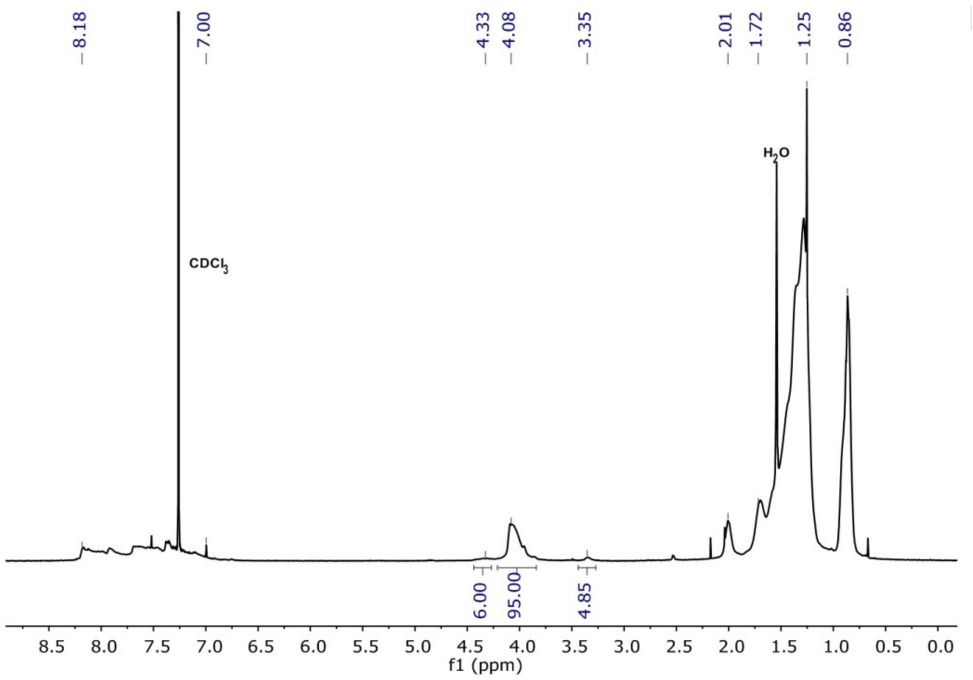

(**c**)

Figure S1. (**a**) ^{1}H NMR spectrum of polymer **P1** in $CDCl_3$; (**b**) ^{1}H NMR spectrum of polymer **P3a** (5% EtPh) in $CDCl_3$; (**c**) ^{1}H NMR spectrum of polymer **P3b** (10% EtPh) in $CDCl_3$.

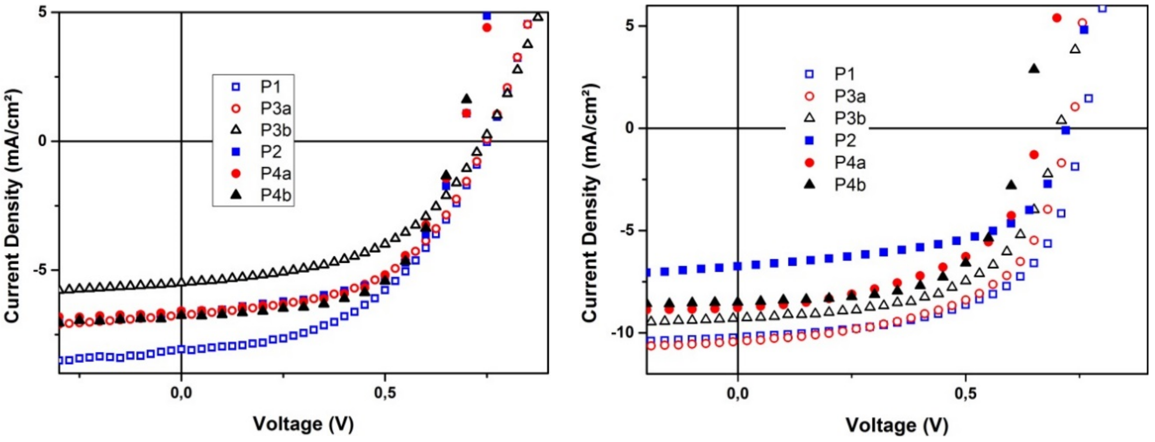

(**a**) (**b**)

Figure S2. *IV* curves for the best PSCs made via RC (**a**) and SC (**b**) using the BT and TzTz based polymer series.

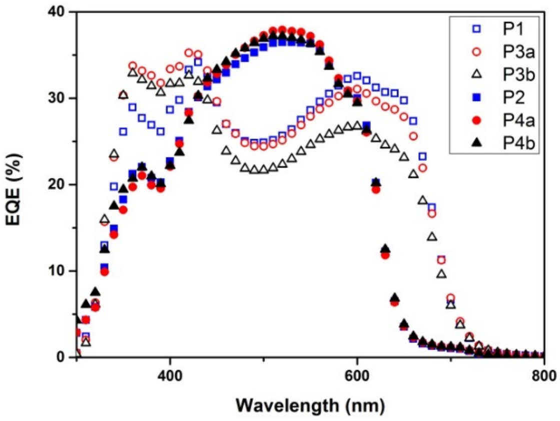

Figure S3. EQE spectra for average RC PSCs using the BT and TzTz based polymer series.

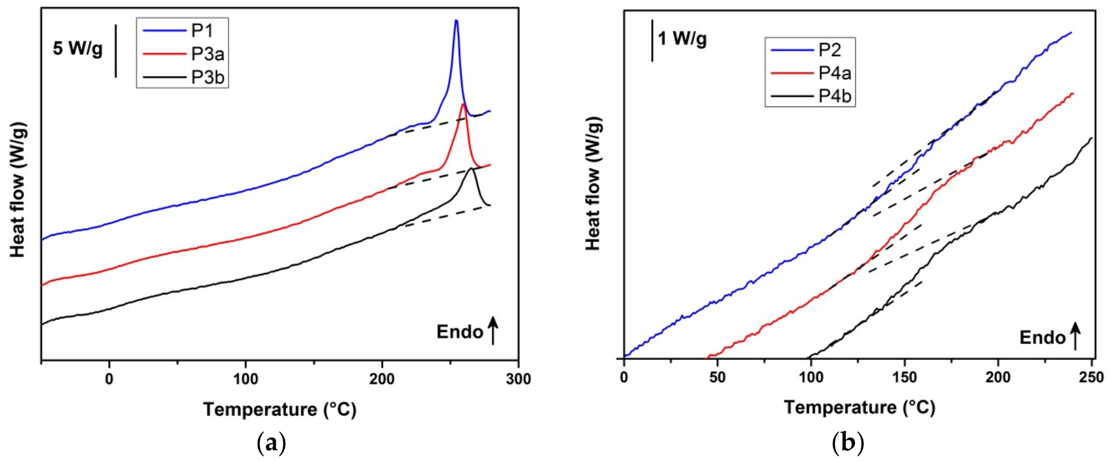

Figure S4. RHC thermograms for the BT (**a**) and TzTz (**b**) based polymers.

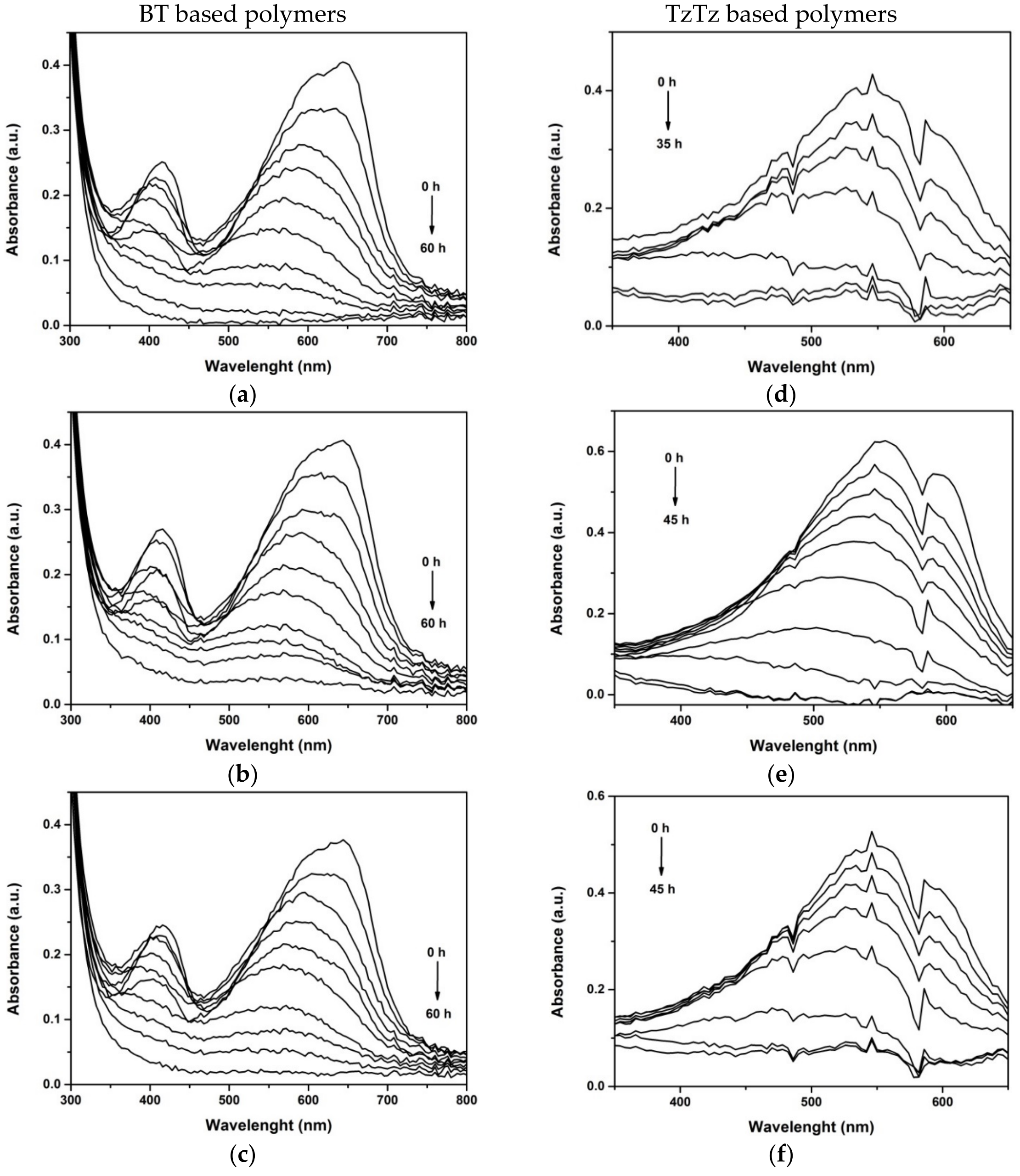

Figure S5. Evolution of the UV-Vis profiles during irradiation of the pristine BT and TzTz based polymer films: **P1** (**a**), **P3a** (**b**), **P3b** (**c**) **P2** (**d**), **P4a** (**e**) and **P4b** (**f**).

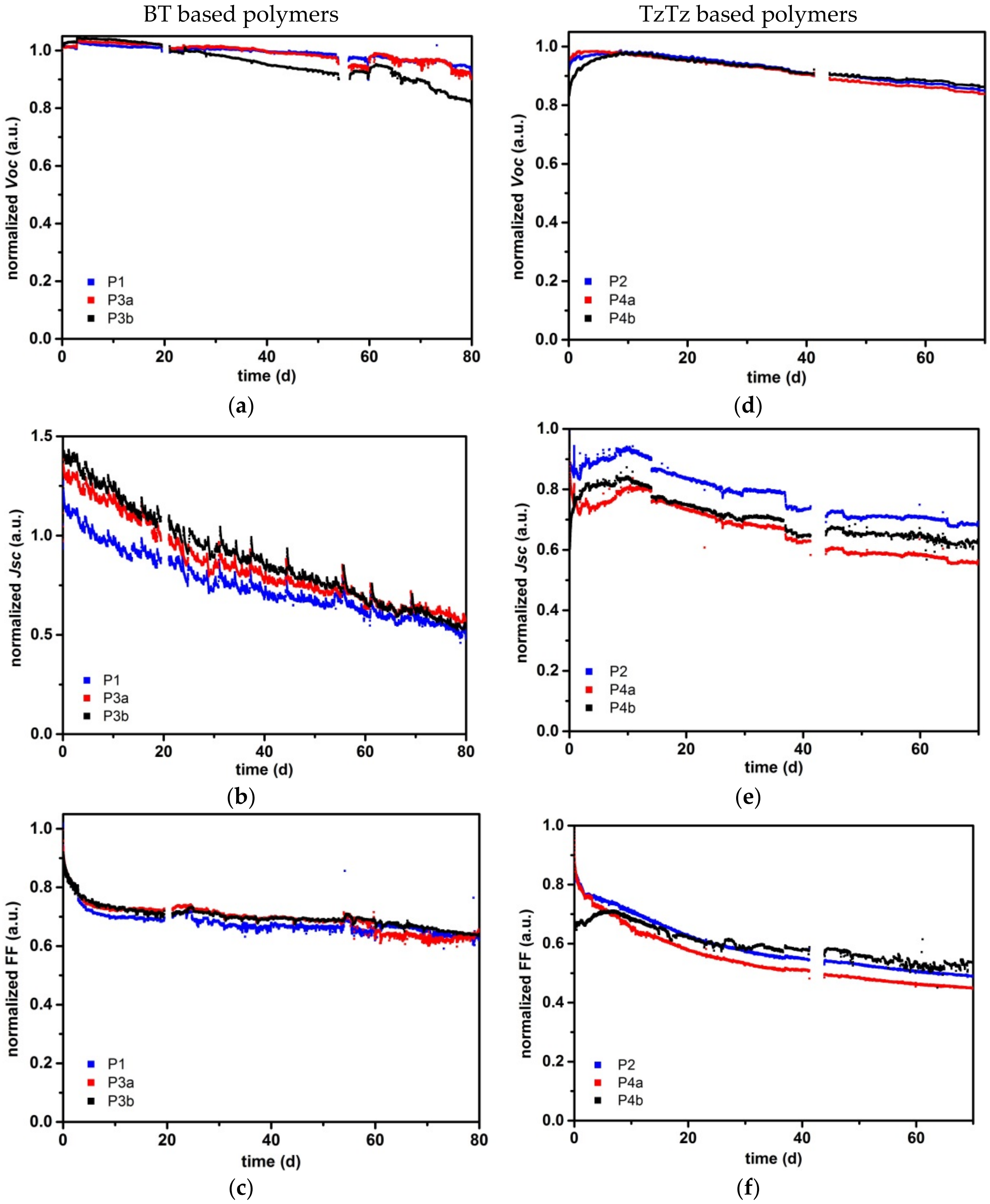

Figure S6. Average lifetime measurements (ISOS-L-1) for the RC devices using the BT and TzTz based polymer series showing the normalized *Voc* (**a**,**d**), *Jsc* (**b**,**e**) and FF (**c**,**f**) trends.

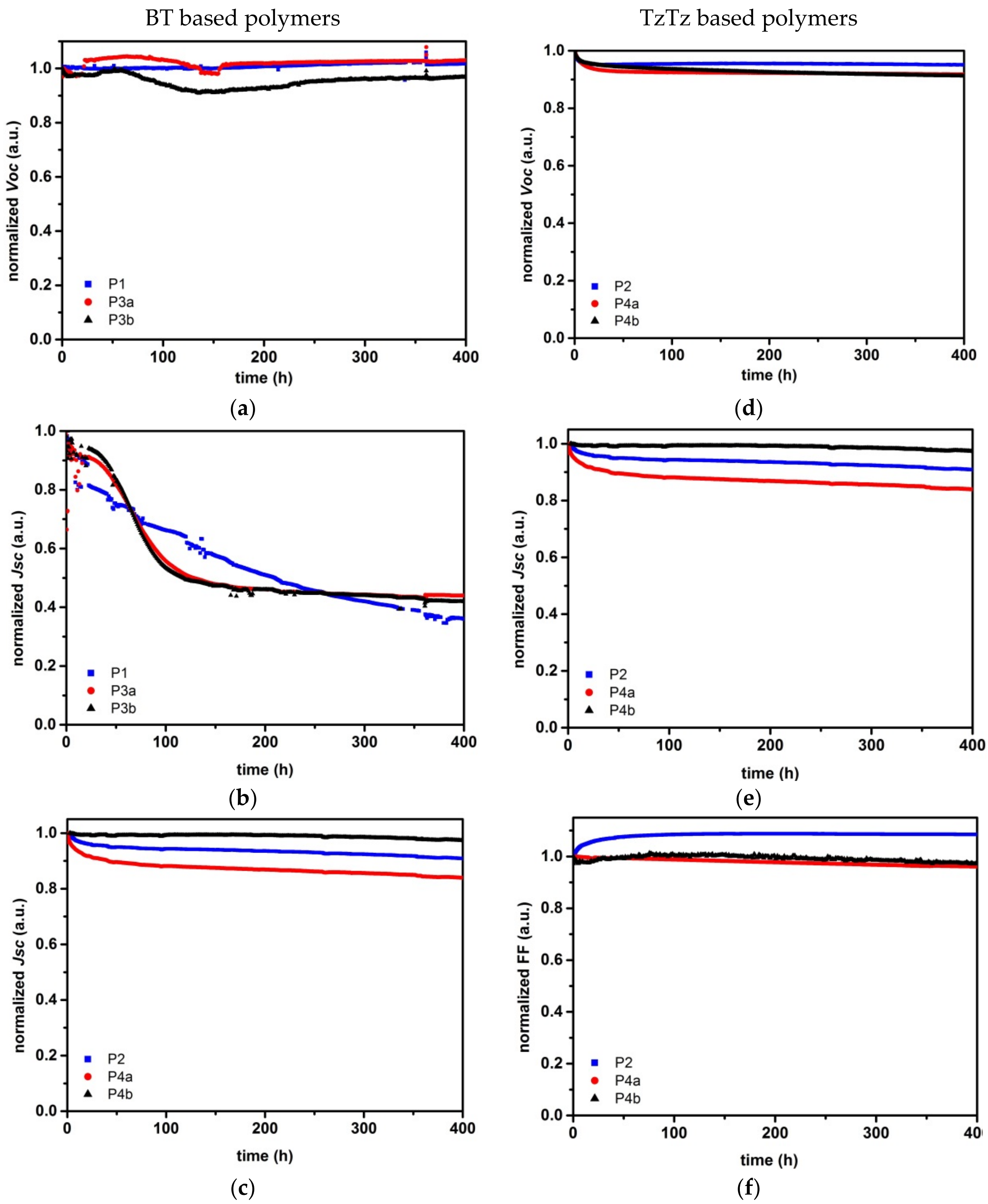

Figure S7. Thermal degradation tests (ISOS-D-2) of the SC devices using the BT and TzTz based polymer series showing the normalized *Voc* (**a**,**d**), *Jsc* (**b**,**e**) and FF (**c**,**f**) trends.

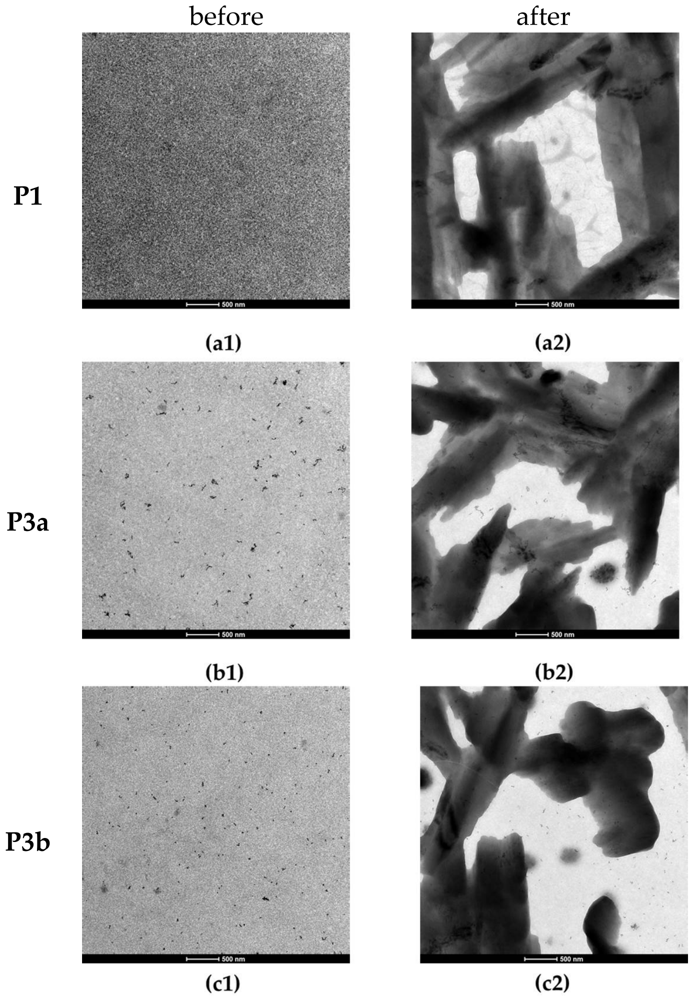

Figure S8. TEM images of the active layers of SC PSCs based on **P1** (**a**), **P3a** (**b**) and **P3b** (**c**) before (1) and after (2) exposure to 85 °C for 400 h.
